# The age and well-being “paradox”: a longitudinal and multidimensional reconsideration

**DOI:** 10.1007/s10433-022-00709-y

**Published:** 2022-05-23

**Authors:** Thomas Hansen, Morten Blekesaune

**Affiliations:** 1 Department of Mental Health and Suicide, Norwegian Institute of Public Health, Oslo, Norway; 2 Oslo Metropolitan University, Oslo, Norway; 3 Promenta Research Center, University of Oslo, Oslo, Norway; 4 University of Agder, Kristiansand, Norway

**Keywords:** Aging, Subjective well-being, Life satisfaction, Affect, Engagement

## Abstract

This paper explores qualifications to the much-discussed paradox that although aging is associated with multiple physical and social losses, subjective well-being (SWB) is stable or increasing in later life. We explore age-related changes in cognitive, affective, and eudaimonic dimensions in three waves of data spanning up to 15 years from the Norwegian NorLAG study (*N* = 4,944, age 40 − 95). We employ fixed-effect models to examine the nature and predictors of aging effects on SWB. Results indicate a general pattern of stability well into older age, but negative changes in advanced age across well-being measures. Declines in SWB are less pronounced and with a later onset for the cognitive compared with the other measures. Loss of health, a partner, and friends are robust predictors of declining SWB. Women report both more negative affect and engagement than men, and these differences increase with age. In conclusion, while increasing SWB from midlife to the mid-70 s attests to the adaptive behaviors and coping resources of young-old adults, the significant downturns in SWB in advanced age point to limits to psychological adjustment when health-related and social threats and constraints intensify.

**Supplementary Information:**

The online version contains supplementary material available at 10.1007/s10433-022-00709-y.

## Introduction

It is a widely held assumption, including by older persons themselves, that subjective well-being (SWB) declines with age (Lacey et al., 2006). Indeed, this expectation seems valid given the many losses and declines that may accompany old age in areas such as roles, energy, income, social relationships, and health. Yet for decades, many researchers claimed that well-being remains stable or increases in later life (Wettstein et al. 2016). For example, meta-studies of up to 132 countries find a U-shaped relationship between age and life satisfaction in most countries, with a minimum level usually occurring between ages 40 and 50 (Blanchflower, 2020; Blanchflower & Oswald 2008). Other studies show little variation in SWB (among adults aged 40 +) (Diener et al. 1999) or that any increase is negligible. For example, a study of World Value Survey data from 64 countries shows only a tiny (one-tenth of a point on the 10-point scale) increase from age 40 to 85 in life satisfaction. Such main trends might conceal important cross-country variation: In 42 of the 69 countries, the correlation with age (above age 45) was negative (Bartram 2020). A handful of longitudinal studies using data from large Western panel surveys corroborate these patterns, showing stable or increasing life satisfaction from middle age up to at least age 70 (Baird et al. 2010; Biermann et al. 2022; Cheng et al. 2017; Frijters & Beatton 2012; Jivraj et al. 2014). The absence of declines in SWB in stages of life when objective life conditions are deteriorating has been labeled a paradox (e.g., Baltes & Baltes 1990; Blanchflower & Oswald 2008).

Several explanations have been advanced to explain this paradox (Hansen & Slagsvold 2012). One addresses the greater use among older adults of accommodative strategies, such as rescaling goals, adjusting aspirations, and downward comparison to worse-off others (George 2006). These strategies help to maintain positive self-evaluations and well-being even in the face of social losses and declining health (Ryff 1991). A related type focuses on gains in competencies to regulate emotional experience. With advancing age, because they sense that time is limited, people increasingly prioritize emotionally meaningful goals and social interactions to maximize positive affect and minimize negative affect (Carstensen 1995). With age, there seems to be an increased favoring of positive over negative stimuli even at the level of attention and memory: older people, more than younger adults, attend to and remember positive information and memories better than negative ones (Carstensen & Mikels 2005). The strategies together with accommodative strategies may explain why older adults seem to have less severe and more short-lived emotional reactions to detrimental life events than younger adults (Beaumont, 2002; Gana et al. 2004). A third explanation addresses adaptational processes and the fact that changing life circumstances often have only small and short-term effects on SWB; over time, SWB tends to fall back to its stable (baseline) level, determined by genes and personality traits (Lucas 2007). A potential fourth explanation could be that older adults tend to exhibit higher levels of psychological characteristics or interpersonal character strengths such as gratitude, compassion, forgiveness, and tolerance, which are linked to a wide range of healthy relational and emotional outcomes (e.g., Beadle & De la Vega 2019; Chopik et al. 2019).

There might be several qualifications to the notion that SWB increases with age, however. Perhaps most importantly, the notion may not hold across dimensions of SWB. SWB is conventionally conceptualized as comprising a cognitive and an affective component (Diener et al. 1999). The cognitive component is usually measured with global evaluations of life satisfaction, whereas the affective well-being encompasses positive affect (e.g., joy, happiness, and calm) and negative affect (e.g., stress, anger, and shame). Over the last two decades, some researchers (e.g., Diener et al. 2010; Vittersø, 2013) and guidelines on measuring SWB (NRC 2013; OECD 2013) include also a third component commonly referred to as eudaimonic well-being. This dimension can be defined and measured in terms of growth, meaning, and interest and engagement (Delle Fave Massimini & Bassi, 2011; Huppert et al. 2009; Huppert & So 2013; OECD 2013; Sheldon 2018; Vittersø, 2013). The “well-being paradox” literature typically focuses on the cognitive component of SWB and ignores the multidimensionality of SWB (Blanchflower, 2020; Hansen & Slagsvold 2012). This is unfortunate as disparate dimensions may have differential relationships with aging (Galambos et al. 2020). For example, while downwards adjustment of comparisons standards may predict positive adjustment and life satisfaction (cognitive well-being), health and social network losses may nevertheless compromise positive emotional experiences (affective well-being) and people’s sense of meaning and engagement (eudaimonic well-being). Indeed, although previous research on the relationship of affective and especially eudaimonic well-being to age is relatively scarce, it tends to indicate a more detrimental change in late life than life satisfaction. For example, a meta-study of mostly US research shows that positive affect tends to decline over the lifespan and negative affect is stable but increasing in later life (Pinquart 2001).

The sparse findings on age trajectories in eudaimonia are less consistent. Using an amalgam measure (CASP-19), some longitudinal studies show a curvilinear relationship whereby scores are higher in the ages 60–79 than in younger and older groups (Steptoe et al. 2012), or a steady decrease with age (Jivraj et al. 2014). Cross-sectional and longitudinal studies on specific subcomponents tend to show accelerating decline in later life for sense of control and purpose in life (Keyes et al. 2002; Mackenzie et al. 2018; Pinquart 2002; Ross & Mirowsky 2002) but a stable or increasing sense of personal meaning (Steger et al. 2009). It is difficult to get a holistic impression of change in SWB, however, as there are few multidimensional studies and one is left with comparing studies that are not directly comparable given differences in age groups, countries, and measures (Jivraj et al. 2014).

The relevant literature has some other gaps which need further investigation. First, as already indicated, the age and well-being paradox may hold only up to a certain age and not in very old age, when psychosocial losses intensify and individuals no longer have the coping resources to maintain high SWB. The opposite argument could also be made, as people in advanced age, following social comparison mechanisms, may feel fortunate and count their blessings simply for being alive. It is thus unfortunate that few studies include the very old (age 80 +) and/or examine nonlinear patterns of change in SWB in older age (Kunzmann et al. 2000). Studies that do, however, tend to show a steep longitudinal decline in life satisfaction in the ages 70 + (Baird et al. 2010; Bittmann 2021; Brockmann 2010; Hansen & Slagsvold 2012; Wunder et al. 2013) and a large drop in life satisfaction in the years before death (Gerstorf et al. 2008; Schilling 2005, 2006). Second, most of the relevant literature relies on cross-sectional data, which can cause systematic distortions of how aging affects SWB. Longitudinal data are needed to avoid (i) conflating age-related change with cohort differences and (ii) selection bias resulting from not accounting for the lower longevity and higher attrition of respondents with lower SWB (Kratz & Brüderl 2021). Longitudinal studies are sparse, especially from a Nordic context. Third, the quadratic specification bias is pervasive as researchers often use a quadratic age specification as “the default” (ibid.). This practice seems influenced by the scholarly focus on the “U-curve,” and rules of the identification of more complex curvilinear relationships.

Fourth, many reports of high late-life SWB are based on analyses that control for socioeconomic variables such as health and marital status (Blanchflower & Oswald 2008; Frijters & Beatton 2012; Jivraj et al. 2014). Some researchers have argued that this procedure is incorrect and makes for counterfactual presentations of the psychological changes that occur when people grow older (Deaton, 2008; Glenn 2009). Bartram (2020) argues that regression models should only control for confounders that are causally prior to both the dependent variable and the core independent variables of interest. For age, there are no individual-level confounding variables to control for, as the “usual suspects” cannot be determinants of age. That said, it can be helpful to include potential intervening variables in separate models (i) to enable identification of the mechanisms that explain patterns of (actual) SWB change and (ii) to assess “pure” aging effects, i.e., genuine direct positive effects of age such as mitigation of unrealistic aspirations that help explain the paradox (Bartram 2020; Kratz & Brüderl 2021).

Fifth, age–SWB relationships may vary across countries with different cultural and institutional frameworks. Cross-national studies show that a U-shaped pattern between life satisfaction and age exists only in richer countries and that life satisfaction decreases with age in poorer countries (Bartram 2020; Deaton 2008; Morgan et al. 2015; Swift et al. 2014). Part of this heterogeneity likely stems from different socioeconomic conditions and welfare regimes. Indeed, loneliness, depression, and dissatisfaction with life are particularly common among older men and especially women in former socialist countries, a pattern that mirrors their comparatively low health and financial satisfaction and high levels of bereavement (Hansen & Slagsvold 2017). Age–SWB relationships may be distinctly positive in the Nordic countries because of more generous pensions and high quality, affordable medical care than in most other countries.

Finally, a largely overlooked issue is whether age–SWB patterns vary by gender. This neglect is unfortunate given the gendered role trajectories and life circumstances of older adults (Pinquart 2001). For example, women are more exposed to widowhood, spousal caregiving, health-related problems, functional disability, and low income (Bunt et al. 2017; Nolen-Hoeksema & Rusting 2000). Such differences may translate into different age-related psychological changes, as demonstrated in a meta-analysis of 300 studies showing that gender differences in well-being are generally small but become more marked in older age as older women report significantly lower SWB than men (Pinquart & Sorensen 2001).

This paper addresses these shortcomings and aims to challenge the “paradox” of high SWB in old age by providing a more nuanced understanding of changes in SWB in the second half of life. Inspired by Seligman’s (2007) conceptualization of well-being as comprising the satisfying, pleasurable, and meaningful life, we provide a longitudinal analysis of age-related change along cognitive (satisfying), affective (pleasurable), and eudaimonic (engaging) aspects of SWB. We investigate age changes in panel data covering 15 years in different aspects of well-being. We compare the well-being trajectories of men and women, and we investigate potential factors that may help explain such well-being trajectories. More specifically, we hypothesize that the loss of health and social resources compromise well-being in old age.

## Methods

### Data and sample

We use three waves of panel data from the Norwegian Life Course, Ageing, and Generation (NorLAG; doi:10. 18712/norlag3_1) study. The first wave of NorLAG comprised representative randomly stratified (by age and sex) samples of adults aged 40–79 from 30 local areas (Veenstra et al. 2021). In all three waves, respondents were interviewed over the phone, after which they completed a self-administered paper questionnaire (Web-based questionnaire with the option to receive a paper version in the third wave). The first wave was collected in 2002/2003 (*n* = 5,559), the second wave in 2007/2008 (including 68% of the participants from the first wave), and the third wave in 2017, when all living participants of previous waves were asked to participate (*n* = 6,099). Response rates for the three telephone interviews were, respectively, 67, 61, and 68 percent, of which approximately 75 percent completed the self-administrated questionnaire. We restrict the analytical sample to individuals aged 40 to 90, excluding the few (*N* = 25) respondents aged > 90 due to issues of representativeness. More specifically, the limited sample size of those aged 90 + creates large margins of error; in addition, participation among the oldest is likely to be strongly skewed toward higher functioning individuals in a survey that is partly Web based (see Limitations). After listwise deletion, the analytical sample ranges from 4,944 to 4,954 respondents (on average 2.2 observations per person) across outcomes.

### Dependent variables

We investigate four dependent variables: life satisfaction, negative affect, positive affect, and engagement. They are all indices with four to six items each, with high values indicating high levels of SWB to facilitate comparisons between the four dimensions. All items were posed in the self-completion questionnaire, except one life satisfaction item and one positive affect item. All indices are standardized (mean values = 0, standard deviations = 1) in the sample and with Cronbach's alpha statistics varying from 0.71 for positive affect to 0.83 for engagement (0.76 for satisfaction and 0.82 for negative affect).

*Life satisfaction* is measured by the Satisfaction With Life Scale (SWLS) (Pavot et al. 1991). The scale comprises four items (e.g., “I am satisfied with my life”) measured on a five-point scale (1 = strongly disagree, 5 = strongly agree). *Negative affect* is measured by a short version of the Positive and Negative Affect Schedule (PANAS) (Watson et al. 1988), which asks to what extent one has felt six negative emotions (worried, upset, scared, irritable, nervous, afraid) during the past two weeks, 1 = very slightly or not at all, 5 = extremely). *Positive affect* is measured with the positive affect subscale of the Center for Epidemiologic Studies Depression (CES-D) scale (Radloff 1977). The CES-D can be conceptualized as measuring a single, higher-order, general depression factor and at a lower level as measuring four specific depressive symptoms factors (Radloff 1977). This four-factor structure has been replicated consistently across a number of studies and different populations and patient groups (see McDowell 2006, for a review), for example, in a meta-analysis of 28 studies (Shafer 2006). One of the factors is positive affect (the others being depressed affect, somatic complaints, and interpersonal problems). Respondents were asked to indicate on a four-point scale (1 = rarely or none of the time, 4 = all of the time) how often they felt happy, hopeful about the future, as good as others, and that they had enjoyed life during the previous week. This four-item positive affect subscale has been used as an emotional well-being indicator in other studies (e.g., Brummett et al. 2009; Gana et al. 2015).

*Engagement* is a core part of the eudaimonic aspect of well-being (Huppert & So 2013). Yet a validated measure or scale on engagement is rarely included in population-based surveys. A novel aspect of the current study is its operationalization of engagement by means of items from the positive affect subscale of the PANAS. NorLAG contains a short version of PANAS that comprises six positive emotions: excited, enthusiastic, alert, inspired, determined, and interested. Respondents were asked to indicate to what extent they have felt these emotions during the past two weeks (1 = very slightly or not at all, 5 = extremely). Although this scale has been used quite extensively as a measure of positive affect (Veenstra et al. 2021), we would argue that its face-value content validity is higher as an indicator of engagement. While the items ignore key aspects of positive affect (e.g., joy and calm), they seem to provide an adequate and broad representation of the eudaimonic concept of engagement.

### Independent variables

The main independent variable is *age*, or (more specifically) aging, as we only present longitudinal findings. *Health* is measured with the physical component of the 12-item Short Form Health Survey (SF-12) (Ware et al. 1996). The variable is divided by 10 to facilitate the interpretation of its impact. We use dummy variables for self-reported *employment status* (done some paid work in the last week), *partnership status* (presence of a cohabiting or married partner in the household), and having a *close friend* (a friend who will be there for them in case of an emergency).

Table 1 presents descriptive statistics of the observations of the sample. The range of the outcome variables indicates that a majority report reasonably high SWB in terms of life satisfaction and positive and (low) negative affect, a phenomenon typically labeled left-biased distribution. Engagement is more balanced. It is worth noticing that such (left) biased distributions tend to be replaced by symmetrical longitudinal distributions in longitudinal models (presented below) because low levels of SWB are typically a time-invariant characteristic picked up by the individual fixed effect. Correlations between dependent variables range from 0.05 (negative affect and engagement) to 0.44 (life satisfaction and positive affect) (others 0.34 − 0.39; results not shown).

**Table 1:** Descriptive statistics of the observations

	Observations	Mean (SD) or %	Range
Life satisfaction	10,971	0.0 (1.0)	− 4.5 − 1.6
Positive affect	10,781	0.0 (1.0)	− 3.2 − 1.1
Negative affect (reversed)	10,874	0.0 (1.0)	− 4.9 − 1.3
Engagement	10,716	0.0 (1.0)	− 3.1 − 2.3
Female	11,213	52.3	0 − 1
Age	11,213	59.7 (10.7)	40 − 90
Partner	11,213	74.6	0 − 1
Close friend	11,213	58.8	0 − 1
Employed	11,213	63.4	0 − 1
Physical health	11,213	4.9 (1.0)	1.1 − 6.7

Furthermore, just above half of the sample were women (52%) and the mean age across the observations was 60 years (*SD* = 11). A majority reported living with a partner (75%), having paid work (63%), having a close friend (59%), and being in relatively good health.

### Analytic strategy

The data are investigated using panel regression models with fixed effects for the individuals. These models provide coefficients indicating how SWB changed “within” people’s life course over the study period (Allison 2009). Our standard model included age as indicated by a linear (centered at 60 years to facilitate interpretations of the coefficients), a quadratic (age^2^), and a cubic term (age^3^). The higher-order terms indicated whether the relationships between age and SWB were linear or nonlinear, and the cubic term can be seen as a test of the U-shaped development in SWB and to capture the theoretically expected dip in later life (Biermann et al. 2022). All aging effects were clearly significant (*p* < 0.001) in statistical tests with three degrees of freedom. The individual fixed effects control (by default) for all time-invariant characteristics of the individuals including cohort. This procedure is recommended when studying aging effects on SWB because it can isolate the effect of age on SWB from cohort effects and mitigate the effect of mortality and selection bias (Beja 2018; Kratz & Brüderl 2021). In separate models, we included controls for time-variant characteristics (health, partnership and employment status, and a close friend) to better understand the predictors and mediators of change in SWB. To test whether aging effects differed between men and women, we investigated interaction terms between gender and linear age.

## Results

**Fig. 1:**
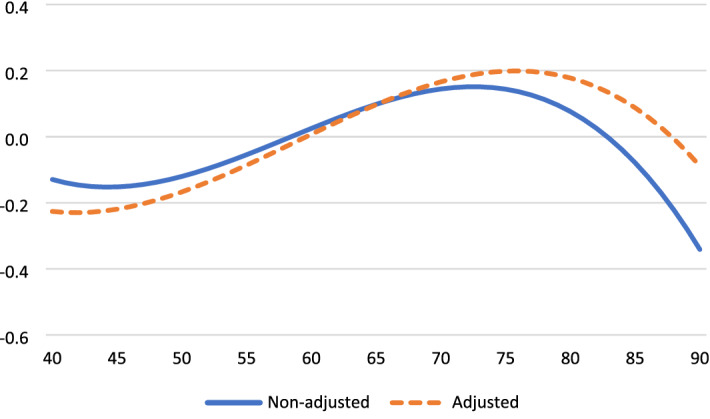
Life satisfaction change from age 40 to age 90

**Fig. 2:**
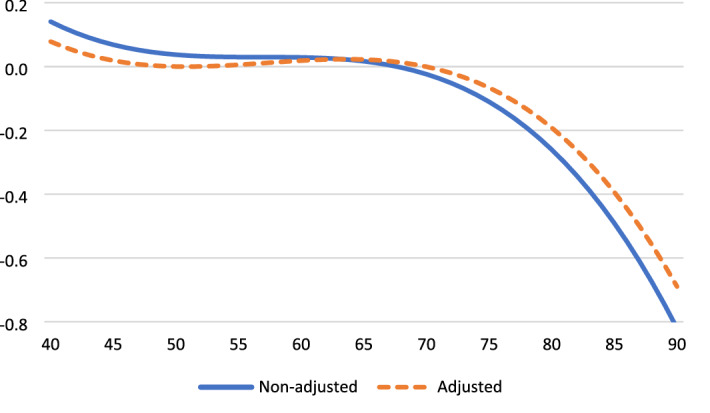
Positive affect change from age 40 to age 90

**Fig. 3:**
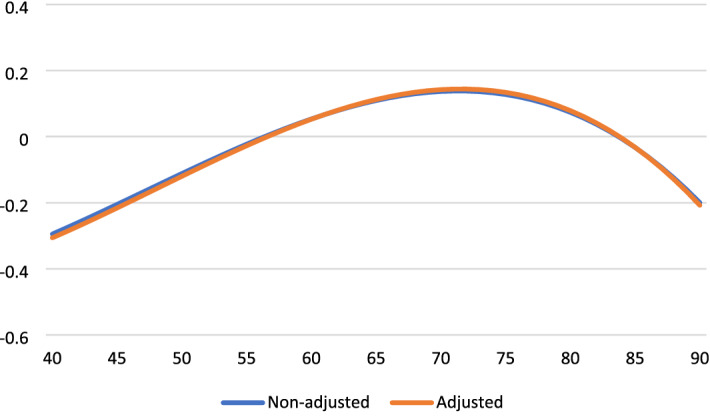
Negative affect (reversed) change from age 40 to age 90

**Fig. 4:**
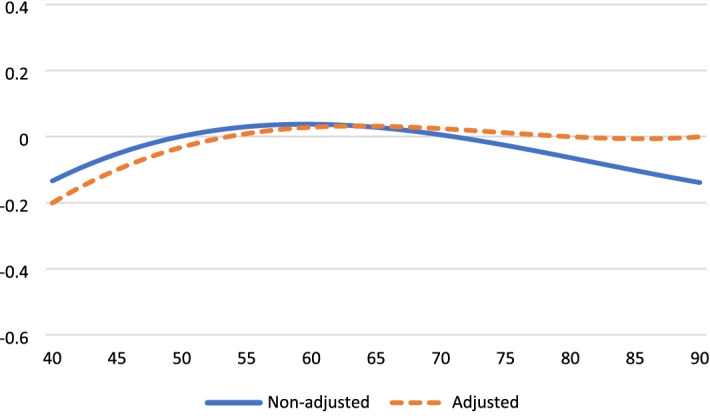
Engagement change from age 40 to age 90

Table 2 presents the results for the analyses of aging effects on four outcome variables, before and after controlling for time-variant factors. These age trajectories are displayed in Fig. 1 through. Life satisfaction increased from the early 40 s, peaked in the mid-70 s, and decreased thereafter (Fig. 1). Life satisfaction dropped by a half standard deviation (*SD*) from age 70 to age 90. Positive affect was relatively stable until age 70 and then dropped strongly (by 0.8 *SD*) to age 90 (Fig. 2). To facilitate comparison across outcomes, negative affect is reversed so high scores indicate for high levels of SWB. SWB as indicated by (low) negative affect improves strongly from 40 to 70 years and deteriorates thereafter (by a third SD to age 90). Fig. 3 Engagement changes less with age than the other three dimensions. Fig. 4It peaks around age 60 and is slightly lower at younger and older ages.

**Table 2:** Fixed-effect (within-person) regression of standardized well-being on age, before and after controls for background variables

	Life satisfaction		Positive affect		Negative affect (reversed)		Engagement	
Age (years-60)/10	0.16 **	− 0.19 **	− 0.01	0.02	0.14 **	0.14 **	0.00	0.02
Age^2^	− 0.01	− 0.02	− 0.02 *	− 0.02 *	− 0.04 **	− 0.04 **	− 0.03 **	− 0.03 **
Age^3^	− 0.03 **	− 0.02 **	− 0.02 **	− 0.02 **	− 0.01 *	− 0.01 *	0.01	0.01
Partner		0.24 **		0.05		− 0.16 **		0.04
Close friend		0.08 **		0.10 **		0.02		0.09 **
Employed		0.01		0.02		0.03		0.01
Physical health		0.11 **		0.06 **		0.04 **		0.07 **
Constant	0.02 **	− 0.75 **	0.03 **	− 0.38 **	0.05 **	− 0.05	0.04 **	− 0.42 **

^*^p < .05, ** *p* < .01

The adjusted aging effects show that time-varying covariates explain some of the age-related changes in satisfaction, engagement, and positive affect, but not in negative affect. In particular, the covariates mediate part of the downturn in SWB after age 70. Life satisfaction is strongly affected by the loss of a partner, somewhat affected by reduced health and the loss of a close friend, but unrelated to the loss of employment. Both positive affect and engagement are negatively affected by the loss of friends and reduced health.

We have in ancillary analyses (displayed in Supplementary Table and Figures) explored interactions between age and *gender*. In models controlling or not controlling for time-variant characteristics (health, partnership and employment status, and a close friend), we find significant (*p* < 0.05) interactions for two of the four dimensions of SWB: negative affect and engagement (Table S1, Figures S1 and S2). As shown, women show a less beneficial development in negative affect compared to men, but a more beneficial development in engagement.

## Discussion

The “paradox” that older people, despite their lower objective quality of life, report higher SWB than younger people, has been subject to much theoretical and empirical attention. A common understanding is that mental strategies help older people to cope with psychosocial losses such that SWB remains or increases (e.g., Kratz & Brüderl 2021). This paper explores potential qualifications to the notion that SWB increases with age. In particular, we were interested in whether the notion holds only for certain dimensions of SWB, up to a certain age, for both genders, and only after control for age-related losses.

Prior work suggests that high SWB in old age is limited to the cognitive component and does not generalize to the affective and eudaimonic components of SWB. In contrast, we find stable or increasing SWB well into old age across all examined components. Particularly for life satisfaction and negative affect SWB improves quite substantially from midlife to early old age, peaks around age 70–75, and deteriorates progressively thereafter. Some researchers have argued that such changes are trivially small (see Blanchflower, 2020). Our findings, however, point to large and practically significant downturns. For example, life satisfaction changes by nearly 0.3 standard deviations and negative affect by 0.4 standard deviations from age 40 to age 70. The size of this increase in group-level SWB is larger than the effect of loss of partner and thus likely highly meaningful.

The fact that SWB is high and increasing well into old age (70 − 75 years) attests to the resources of individuals to sustain a sense of purpose and well-being, even in the face of age-related risks of physical deterioration and other stressors. The maintenance of SWB in later life is typically assumed to derive from adaptation, emotional regulation, and accommodative strategies such as rescaling of goals and adjusting aspirations (Carstensen et al. 1999; George 2006). Whether interpreted as a “gain” or simply as “resignation,” reductions in comparison standards and aspirations seem adaptive to maintain a sense of well-being in later life (Hansen & Slagsvold 2012).

Our findings show declining well-being after age 70–75 for three of the four SWB dimensions (not for engagement). A marked decline in SWB in late life, especially in the last stages of life, has been show previously (e.g., Baird et al. 2010; Bittman 2021). Notwithstanding high heterogeneity, these patterns suggest that the oldest old have a distinct and less desirable social and physical profile that causes accelerated decline in SWB. Individuals may lack the coping resources or human interaction to sustain a positive outlook and resilience when uncontrollable and pervasive psychosocial stressors accumulate or intensify. It should be noted that the observed drops in SWB appear substantial and important. For example, the size of the declines in cognitive and affective outcomes from the high point in the mid-70 s to age 90 range from 0.3 to 0.8 standard deviations. That said, it should also be recognized that the levels of well-being among older Norwegians (e.g., mean SWLS score of 16 out of maximum 20, or mean life satisfaction of 7.5 on a 0 − 10 scale (supplementary analysis)) are exceptionally high compared to any age group in most other Western countries (Sachs et al. 2018).

There is the possibility that positive age-related SWB developments are confined to countries such as Norway, with relatively high life expectancy and generous welfare support for older adults (Hansen & Slagsvold 2017; Steptoe et al. 2015). While we were unable to test this question directly, a rough comparison with similar longitudinal studies from other countries seems to show similar patterns of late-life reductions in SWB (e.g., Kratz & Brüderl 2021; Steptoe et al. 2012). Although the mean level of SWB is higher and the point at which it starts to decline is somewhat postponed compared to in data from countries such as Germany and the UK (ibid.), both this and other studies demonstrate that the final stage of life is associated with significant drops in quality of life irrespective of cultural and institutional frameworks.

Overall findings reveal similar age-related changes in SWB for men and women. Still, significant age-by-gender interactions are evident for negative affect and engagement, suggesting that women report more of both experiences compared with men and that gender differences increase with age. A male advantage at older ages has been shown previously for cognitive and affective outcomes (Hansen & Slagsvold 2017; Pinquart 2001). Age-related changes and gender differences thereof regarding engagement are relatively unresearched. We hypothesize, although speculatively, that women’s higher levels of both negative affect and engagement in later life could reflect their generally greater involvement in the lives of social network members and in caregiving roles (Pinquart & Sorensen 2001). While potentially fostering greater emotional involvement, focused effort, and absorption (i.e., engagement), it may also cause more concern, worries, and upset (i.e., negative affect).

The broader age-related patterns (stable or improved SWB until about age 70) are rather similar before and after introducing controls for common psychosocial and health-related changes in later life. The controls variables with quite consistent positive impacts across outcomes are physical health and having a partner and/or close friend. Employment status has no statistically significant effect. Hence, the “paradox” of well-being in old age seems to hold only in early old age, but irrespective of the introduction of controls. Still, the late-life drops in SWB become less pronounced in the ceteris-paribus approach. While especially change in partnership status and physical health mediate some of the aging effects, there should be age-related factors unaccounted for that can explain late-life decline in SWB. Possible candidates may be other aspects of social (e.g., social participation and loneliness) and health-related (e.g., mobility, pain, and sleep) status or functioning. It is known, also, that compared with affective well-being, cognitive well-being correlates more strongly with goal- or status-related factors (e.g., unemployment and income), whereas the opposite holds for factors influencing how we spend our time (e.g., health status and close social ties) (e.g., Kahneman & Deaton 2010). While our selection of controls seem quite balanced in this respect, for instance, *partner* and *employed* could be predicted to correlate more with life satisfaction and *physical health* and *close friend* more with affect variables, findings suggest that the controls generally correlate more strongly with life satisfaction. We thus may not capture well some of the key factors mediating the age–affect associations.

This study has some other limitations but also strengths. One strongpoint is the analysis of change “within” older people’s lives. This method reduces the so-called selection of relative resourceful individuals among the oldest old, which tend to bias results based on cross-sectional data (Kratz & Brüderl 2021). A related strength is the use of age polynomial models that also include a cubic term, which allows for more flexibility in the study of aging effects and avoids quadratic specification bias that tends to produce u-curves by only including linear and squared terms (Biermann et al. 2022). However, while our statistical models control for cohort variation and other fixed characteristics of the individuals, we were unable to correct for period effects because of colinearity with the aging effects. Hence, our results merely indicate how SWB changed in our study population in a certain historical period. For robustness, these results should be compared with other populations and other periods. Other strengths include a broad scope of dependent variables, the use of large-scale and long-running panel data, and assessments on the very old (up to age 90). A further strongpoint is the reliance on self-completion questionnaires for dependent variables, which should mitigate social desirability bias and improve reliability when probing sensitive issues such as SWB (Hansen & Slagsvold 2016). At the same time, however, these methods may exacerbate issues relating to sample selection bias. While the oldest respondents of our sample (age 90) are older than in most previous studies, the sample is biased toward the non-frail and the higher educated, and it excludes institutionalized persons (Veenstra et al. 2021). These biases may be amplified by using self-administered questionnaires, in particular the use of online questionnaires in wave three. Furthermore, we are limited in our indicators of eudaimonic well-being. It would be interesting to explore aging effects on existential measures such as the experience of meaning, purpose and direction in life, and growth and development. As discussed, it would also be interesting to explore these effects in countries with lower life expectancy and less comprehensive welfare provisions than in Norway. Finally, both potential strength and weakness of our study are the use of PANAS to assess eudaimonic well-being. We have argued that the PANAS items poorly cover the breath of the concept and ignore core positive emotions such as calm, joy, and happiness (see Diener et al. 2010, for a similar critique). Furthermore, some items assess states that are not true feelings (e.g., “determined” and “alert”; ibid.) and seem more like eudaimonic experiences or motivational states. We have thus argued that the items seem to provide a more adequate and broad representation of the eudaimonic concept of engagement. Few survey data include a standardized measure of engagement. (An exception is a three-item version included in the 2012 European Social Survey.) While we have demonstrated and proposed a novel operationalization of a key eudaimonic concept with a scale that is reliable and routinely included in quantitative surveys, more research is needed to substantiate the validity of this procedure.

In conclusion, this study shows that transitions from midlife to the golden age and into very old age are accompanied by substantial multidimensional shifts in subjective well-being. Happiness or subjective well-being can be conceived of as consisting of three distinct elements: the pleasant life, the good life, and the engaged (or meaningful) life (Diener & Biswas-Diener 2008; Seligman 2002). This study shows that aging is associated with stability or increasing SWB along these three dimensions from middle age and well into older age. However, while even the last phase of life appears to be associated with experiences and events that augment SWB, SWB is multidimensionally and progressively decreasing after age 70. This observation reflects the influence of inevitable challenges and points to limits to psychological adjustment in very old age.

## Supplementary Information

Below is the link to the electronic supplementary material.

Supplementary file1 (PDF 377 KB)

## Data Availability

It can be downloaded free of charge from nsd.no.

## Code availability

N.A.
