# Supplementary material for: The age and well-being “paradox”: a longitudinal and multidimensional reconsideration
Source: Eur J Ageing. 2022 May 23;19(4):1277–86. doi: 10.1007/s10433-022-00709-y (PMC9729496; doi:10.1007/s10433-022-00709-y)
Supplement: Supplementary file 1 — Supplementary file1 (PDF 377 KB) [file 10433_2022_709_MOESM1_ESM.pdf]

Supplementary table and figures

Table S1. Fixed effect (within-person) regression of standardized well-being on age, before and after controls for background variables with interaction terms between linear age and gender.

	Life satisfaction		Positive affect		Negative affect (reversed)		Engagement	
Age (years-60)/10	0.18 **	0.20 **	0.01	0.03	0.16 **	0.17 **	-0.03	-0.01
Age^2	-0.01	-0.01	-0.02 *	-0.02 *	-0.04 **	-0.04 **	-0.03 **	-0.03 **
Age^3	-0.03 **	-0.02 **	-0.02 **	-0.02 **	-0.01 *	-0.01 *	0.00	0.01
Age * female	-0.04	-0.03	-0.03	-0.02	-0.06 *	-0.06 *	0.05 *	0.06 *
Partner		0.24 **		0.05		-0.16 **		0.05
Close friend		0.08 **		0.10 **		0.02		0.10 **
Employed		0.01		0.02		0.02		0.02
Physical health		0.11 **		0.06 **		0.04 **		0.07 **
Constant	0.02 **	-0.74 **	0.03 **	-0.38 **	0.05 **	-0.05	0.04 **	-0.42 **

* p< .05, ** p< .01

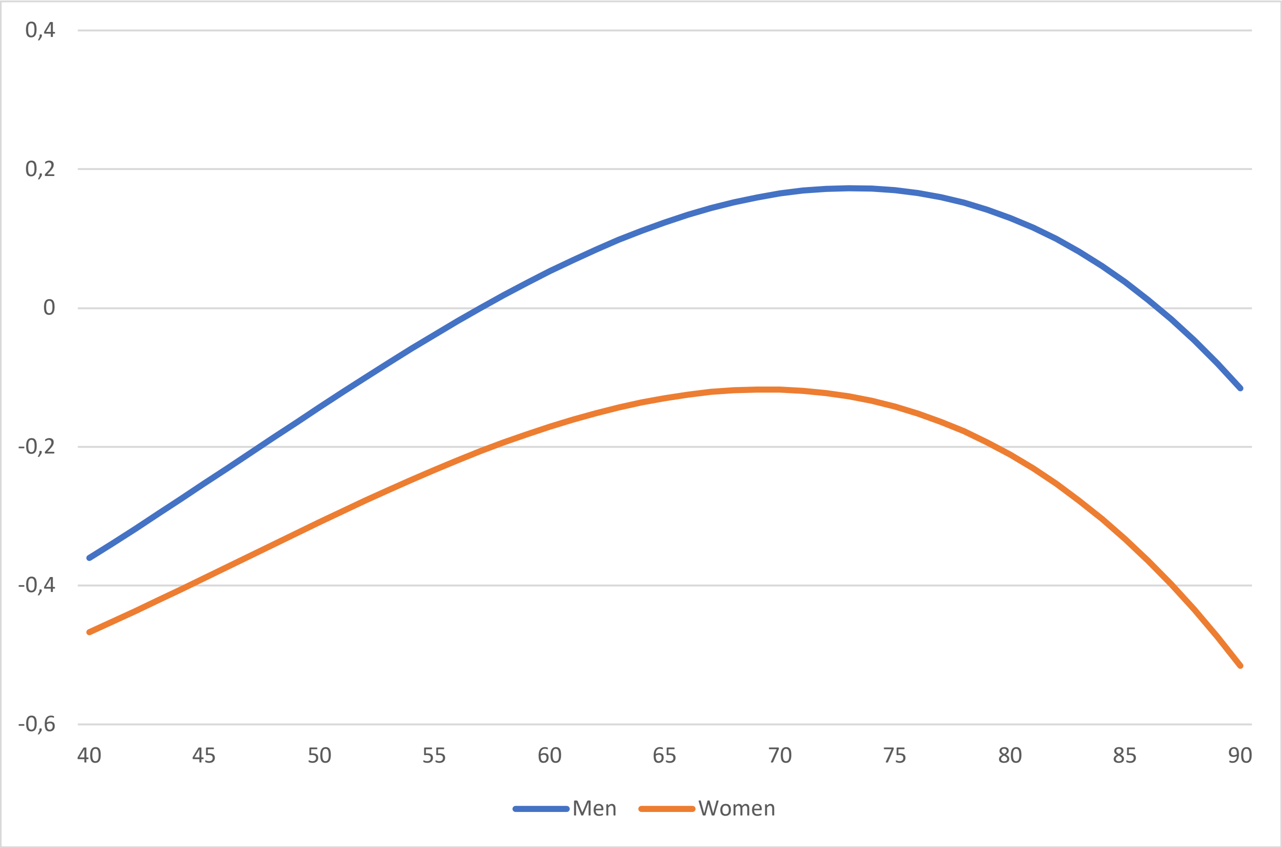

Figure 1: Negative affect (PANAS) related to aging (40-90 years) by gender

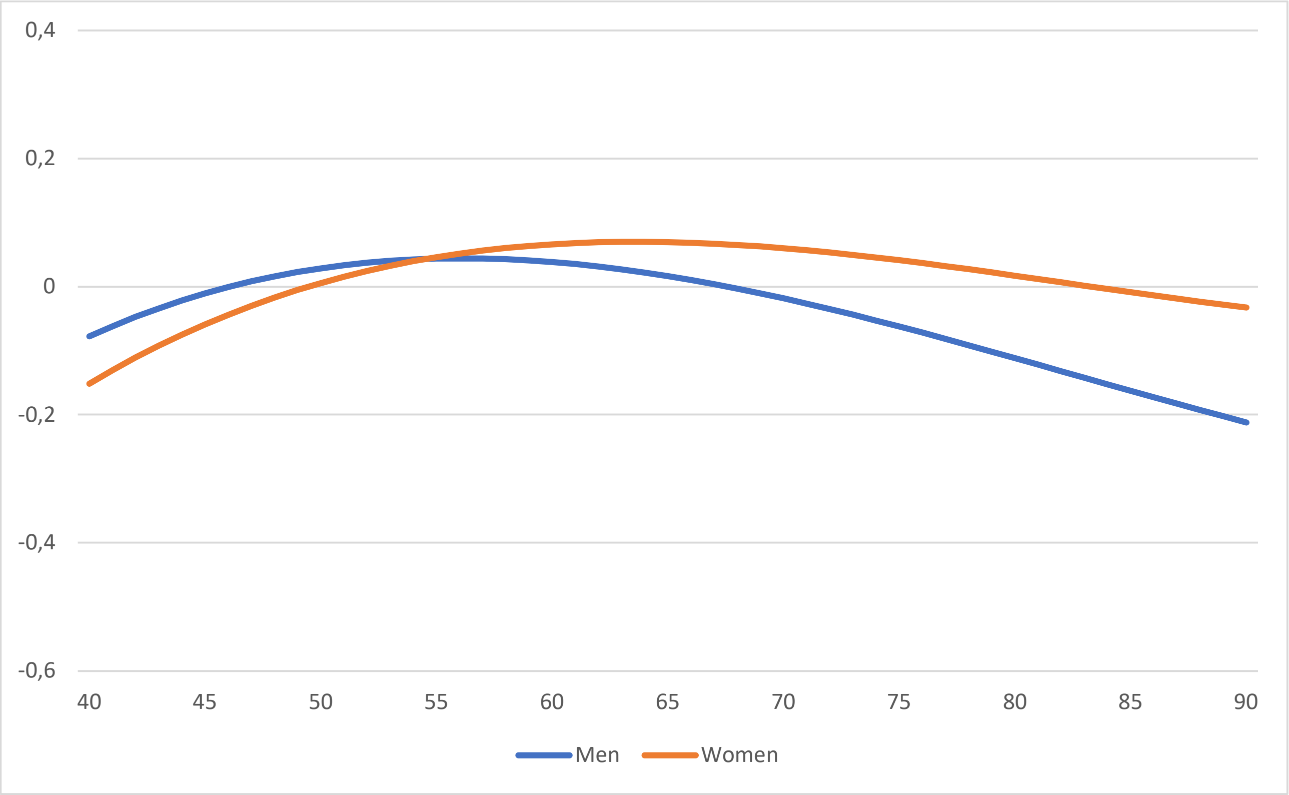

Figure 1: Engagement related to aging (40-90 years) by gender
